# *Panax ginseng* Modulates Cytokines in Bone Marrow Toxicity and Myelopoiesis: Ginsenoside Rg1 Partially Supports Myelopoiesis

**DOI:** 10.1371/journal.pone.0033733

**Published:** 2012-04-16

**Authors:** Hanumantha Rao Balaji Raghavendran, Rekha Sathyanath, JangWoo Shin, Hyeong Keug Kim, Jong Min Han, JungHyo Cho, Chang Gue Son

**Affiliations:** Liver and Immunology Research Center, Daejeon Oriental Hospital Daejeon, University, Daejeon, Republic of Korea; National Institutes of Health, United States of America

## Abstract

In this study, we have demonstrated that Korean *Panax ginseng* (KG) significantly enhances myelopoiesis *in vitro* and reconstitutes bone marrow after 5-flurouracil-induced (5FU) myelosuppression in mice. KG promoted total white blood cell, lymphocyte, neutrophil and platelet counts and improved body weight, spleen weight, and thymus weight. The number of CFU-GM in bone marrow cells of mice and serum levels of IL-3 and GM-CSF were significantly improved after KG treatment. KG induced significant c-Kit, SCF and IL-1 mRNA expression in spleen. Moreover, treatment with KG led to marked improvements in 5FU-induced histopathological changes in bone marrow and spleen, and partial suppression of thymus damage. The levels of IL-3 and GM-CSF in cultured bone marrow cells after 24 h stimulation with KG were considerably increased. The mechanism underlying promotion of myelopoiesis by KG was assessed by monitoring gene expression at two time-points of 4 and 8 h. Treatment with Rg1 (0.5, 1 and 1.5 µmol) specifically enhanced c-Kit, IL-6 and TNF-α mRNA expression in cultured bone marrow cells. Our results collectively suggest that the anti-myelotoxicity activity and promotion of myelopoiesis by KG are mediated through cytokines. Moreover, the ginsenoside, Rg1, supports the role of KG in myelopoiesis to some extent.

## Introduction

Blood/bone marrow system one of the largest organs in the body, is an important potential target of chemical exposure, in particular, chemotherapeutic agents and ionizing radiation. Consequently, analysis of blood and bone marrow has become a routine procedure in the investigation of hematological and bone marrow disorders in chemotherapy and radiotherapy assessments [1], [2]. 5-Fluorouracil (5FU), a pyrimidine analog, is an active chemotherapy drug that is widely used to treat a variety of tumors, including colorectal, breast, and liver carcinomas [3]. However, myelosuppression after 5FU application is a major limiting factor in the clinical treatment of cancers. Toxicity has profound consequences on patients, affecting not only their therapeutic options but also overall quality of life, and restricts the extensive clinical application of 5FU. Notably, 5FU-induced side-effects often lead to discontinuation of treatment [4]. An earlier study showed that ∼40–47% of patients receiving protracted venous infusion of 5FU (300 mg/m^2^/day) for two years required delay in treatment and/or dose reduction due to fatigue and a decrease in the number of blood and bone marrow cells [5].

Ginseng, the root of *Panax ginseng* C.A. Meyer, is traditionally used as a restorative, anti-diabetic, anti-vomiting, and anti-cancer agent worldwide. The major active ingredient responsible for the actions of *Panax ginseng* is ginsenoside, a four-ring, steroid-like structure with sugar moieties [6]. Traditional clinical practice recommends prescriptions containing ginseng in conjunction with chemotherapy to reduce the side-effects of anti-cancer drugs [7]. A recent study showed that the acidic polysaccharide of ginseng is a candidate therapeutic agent with radioprotective activity and enhances the number of hematopoietic and immune cells (bone marrow and spleen cells) in irradiated mice [8]. Another study demonstrated protective effects of total saponins from stem and leaf of *Panax ginseng* against cyclophosphamide-induced genotoxicity and apoptosis in mouse bone marrow cells and peripheral lymphocyte cells [9]. Ginsenosides, which generally constitute one of the major active components of ginseng, are amphiphilic in nature and display the ability to intercalate into the plasma membrane. There is evidence to suggest that ginsenosides interact directly with specific membrane proteins. The ginsenoside, Rg1, enhances *ex vivo* bone marrow stromal cell proliferation to a significant extent in a dose-dependent manner. Since bone marrow stromal cells (BMSCs) play important roles in hemopoiesis and regulating immunoreactivity, the ability of Rg1 to enhance BMSC proliferation is of potential value for clinical applications [10]. Previous research has additionally shown that Rg1 has estrogen-like properties and exerts its action via activation of estrogen receptor alpha in MCF-7 cells [11]. As Rg1 can interact with the glucocorticoid receptor, binding to hormone receptors promotes the recruitment of immature and mature granulocytes from the bone marrow reservoir and prolongs the life-span of mature granulocytes by lowering susceptibility to apoptosis [12].

The mechanism underlying the protective role of Korean ginseng against 5FU-induced myelotoxicity and identification of the active component that supports its action during myelopoiesis remain significant challenges in cancer treatment. In the present study, we have focused on the immunogenic activity of KG against 5FU-induced myelosuppression in mice and bone marrow cells cultured *in vitro*, as well as the role of the predominant component, ginsenoside Rg1, in myelopoiesis *in vitro*.

## Results

### Effects of KG on 5FU-induced hematotoxicity and loss of body weight

**Figure 1:**
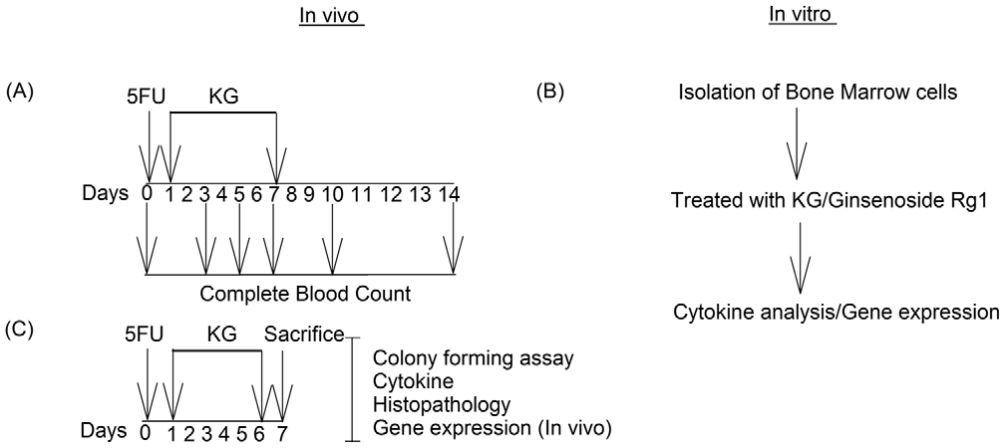
Invivo experimental design (A), C57BL6 (n = 40) were treated with 5FU (200 mg/kg, i.p) on day zero and post-treated (24 h) with KG (25, 50 and 100 mg/kg) for six days, daily once. Complete blood count (CBC) was taken at 0, 3, 5, 7 10 and 14 day. C57BL6 mice (n = 40) were treated with 5FU (200 mg/kg, i.p) on day zero and post-treated (24 h) with KG (25, 50 and 100 mg/kg) for six days, sacrificed on day 7 for cytokines, histopathology, gene expression and colony forming studies (B). Bone marrow cells isolated from C57BL6 were cultured and treated with KG (0.2, 2, 20 and 200 µg/ml) or Rg1 (0, 0.5, 1 and 1.5 µmol/L) at for cytokines (ELISA) and gene expression by semi quantitative and quantitative PCR (C).

**Figure 2:**
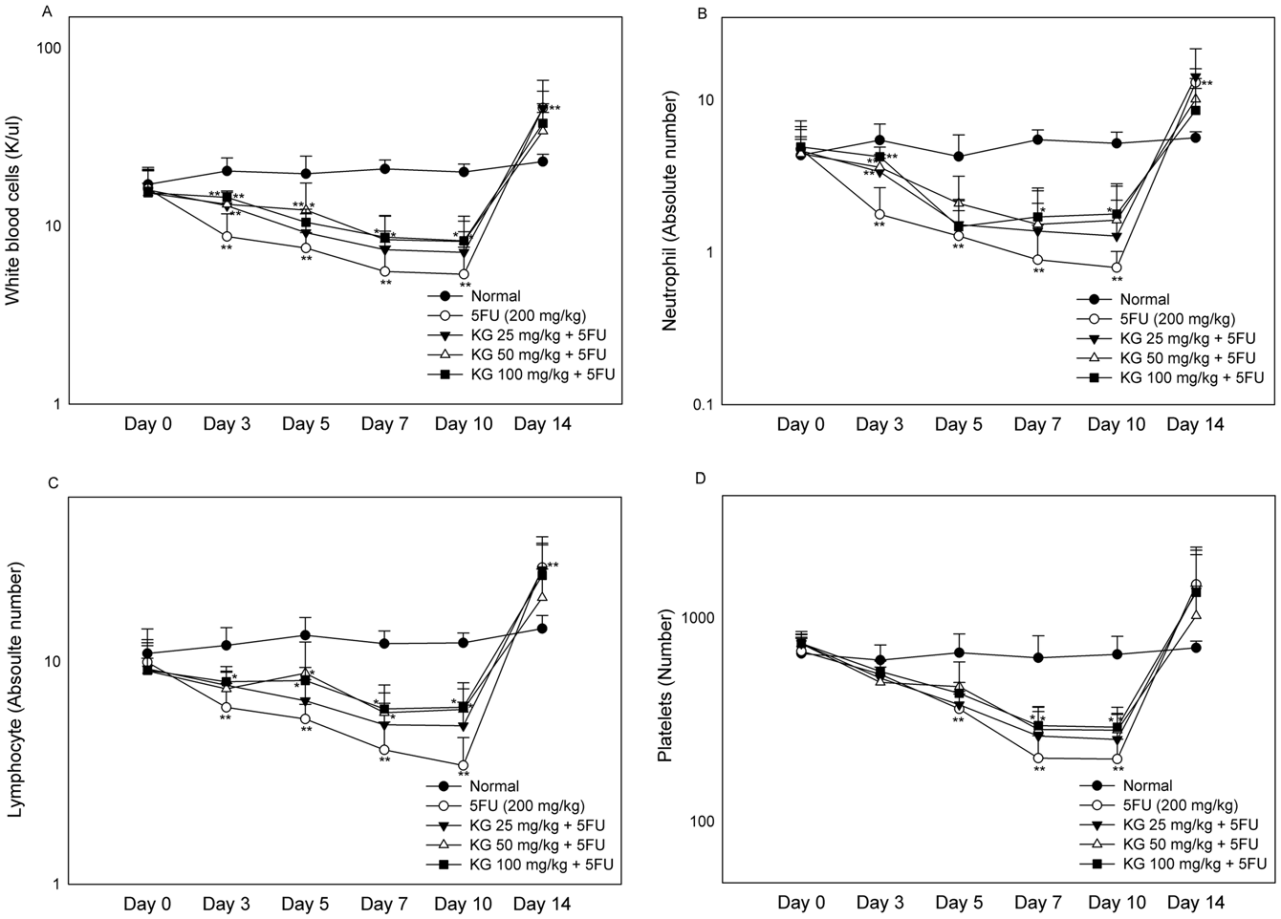
Effects of KG on Haematologic parameters in 5FU treated mice. Before and after 24 h of 5FU injection and KG treatment, about 60–100 µl of retro orbital sinus blood was collected using heparin coated capillary tube at different days (0, 3, 5 7 10 and 14) and haematological parameters such as (A) white blood cells (WBC), (B) neutrophils, (C) lymphocytes (absolute number) and (D) platelets were measured. Data were expressed as means ± SD (*n* = 8). ^*^ *P*<0.05 ^**^ *P*<0.01, normal Vs. 5FU and KG Vs. 5FU.

**Figure 3:**
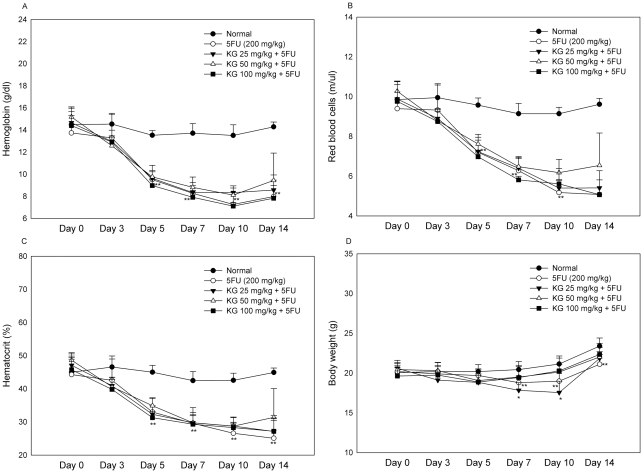
Effects of KG on Haematologic parameters and body weight changes in 5FU treated mice. Before and after 24 h of 5FU injection and KG treatment, about 60–100 µl of retro orbital sinus blood was collected using heparin coated capillary tube at different days (0, 3, 5 7 10 and 14) parameters such as (A) hemoglobin, (B) red blood cells (RBC), (C) hematocrit and (D) body weight were measured. Data were expressed as means ± SD (*n* = 8). ^*^ *P*<0.05 ^**^ *P*<0.01, normal Vs. 5FU and KG Vs. 5FU.

Initially, we examined the protective effects of KG (25, 50 and 100 mg/kg) against the toxic effects of 5FU on white blood cell (WBC), lymphocyte, neutrophil, platelet, Hb and red blood cell counts as well as hematocrit levels in the peripheral blood of mice at different time-points (0, 3, 5, 7, 10 and 14 days). The experimental design for the in vitro and in vivo studies was shown in Fig. 1A, B, C. Leucopenia (Fig. 2A), neutropenia (Fig. 2B), lymphocytopenia (Fig. 2C) and thrombocytopenia (Fig. 2D) were apparent 3 days after 5FU injection. As shown in Fig. 3A, B, C, peripheral anemia, erythrocytopenia and decline in the hematocrit level were evident 5 days after a single intraperitoneal dose of 5FU (200 mg/kg). Recovery of these parameters began after day 11. Significant body weight loss was observed on days 5, 7 and 10 in mice treated with 5FU (Fig. 3D). A rebound reaction was observed for WBC, lymphocyte, neutrohil, and platelet counts on day 14 (Fig. 3A).

Mice subsequently treated with KG (50 and 100 mg/kg) displayed improvements in leucopenia and lymphocytopenia on days 3, 5, 7 and 10. Treatment with 50 mg/kg KG prevented early neutropenia, whereas 100 mg/kg KG prevented neutropenia on days 3, 7 and 10. Thrombocytopenia on days 7 and 10 was improved in mice administered KG (50 and 100 mg/kg), compared to those treated with 5FU alone. Improvement of anemia, erythrocytopenia, and decline in hematocrit after KG treatment were not statistically significant, compared with the 5FU-induced group. The group of mice treated with the lowest dose of KG (25 mg/kg) displayed improvements in leucopenia and neutropenia on day 3. The optimal effect of KG on 5FU-induced hematotoxicity was evident on day 7 after 5FU administration. A significant decline in body weight was observed on days 7 and 10 in mice treated with the lowest dose of KG (25 mg/kg), but not those treated with higher doses (50 and 100 mg/kg) of KG. In our experiments, 200 mg/kg 5FU did not induce lethality, and a similar hematological pattern was observed in the group of mice sacrificed on day 7 (data not shown).

### Effects of KG on 5FU-induced histopathology of bone marrow, spleen and thymus

**Figure 4:**
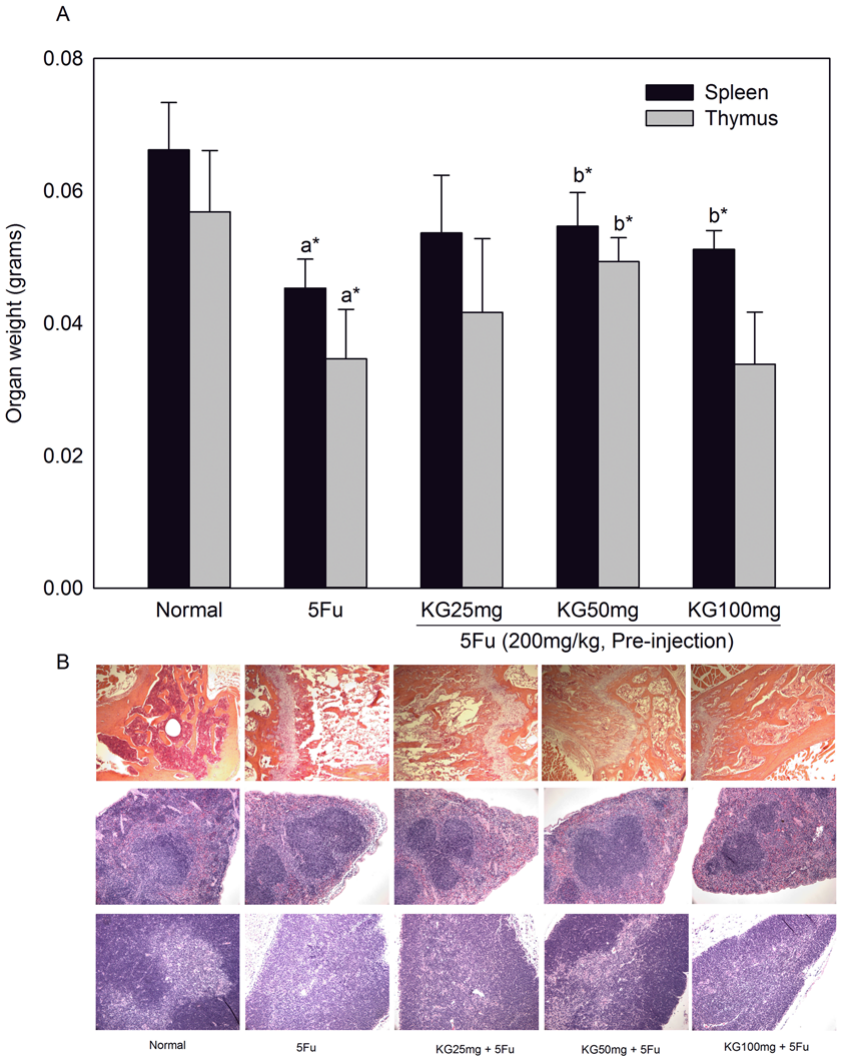
Effects of KG on Organ weight and Histopathology. After 24 h of 5FU injection and 6 days of KG treatment, group of mice were sacrificed under ether anesthesia on day 7 and organ weights such as spleen and thymus were measured using an automated electronic balance (A) Organs such as spleen, thymus and bone marrow were removed and processed for Haematoxylin and Eosin (B). The stained tissues were examined under a light microscope (200X magnifications). Data were expressed as means ± SD. ^*^ *P*<0.05 ^**^ *P*<0.01, a – normal Vs. 5FU, b – KG Vs. 5FU.

The weights of organs, such as spleen and thymus, were significantly reduced after 5FU administration. KG (50 mg/kg) improved spleen weight alone, while KG (100 mg/kg) improved both spleen and thymus weights (Fig. 4A). The bone marrow histology results obtained from mice exposed to high doses of 5FU and KG are shown in Fig. 4B. In contrast to untreated control mice (Normal), 5FU and KG-treated mice showed bone marrow cytotoxicity, hypocellularity and appearance of' adipocytes. Interestingly, these morphological changes were significant in 5FU-treated mice, while KG (50 and 100 mg/kg)-treated mice showed moderate hypercellularity and resistance to 5FU-induced cytotoxicity (Fig. 4B). Spleen tissue histology after 5FU treatment revealed significant hypocellularity in the white and red pulp regions and destruction of capsule. These features were distinctly alleviated after KG treatment, particularly at doses of 50 and 100 mg/kg. Normal thymus is composed of sheets of small and medium lymphocytes, and exhibits normal cellularity. 5FU injection into mice resulted in altered thymic architecture characterized by hypocellularity and loss and pale staining of lymphocytes. Post-treatment with KG led to moderate protection against hypocellularity in the cortex and medulla regions.

### Effects of KG on 5FU-induced myelopoiesis

**Figure 5:**
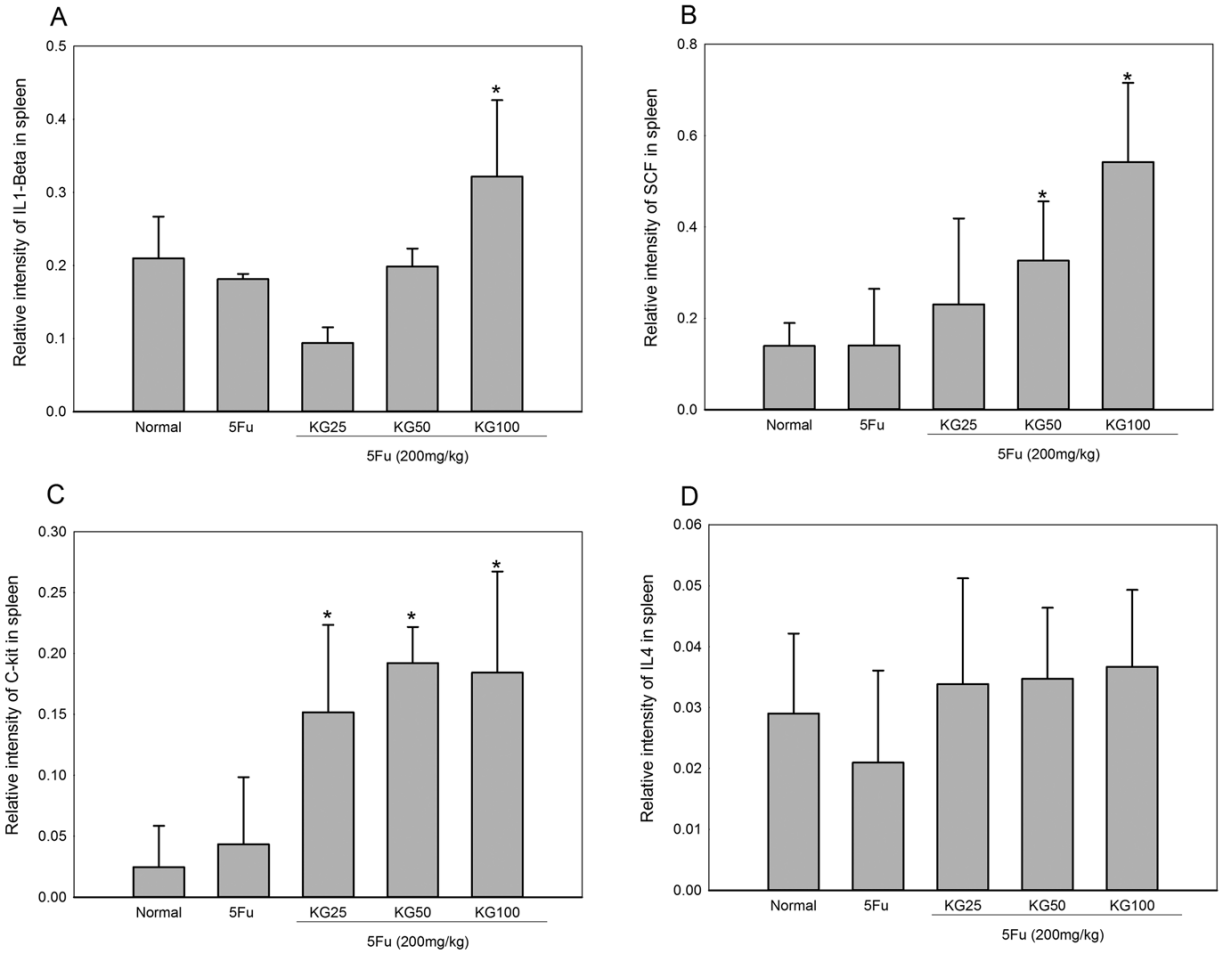
Effects of KG on 5FU-induced changes gene expression in spleen. Total RNA was extracted from the spleen cells, mRNA of (A) IL-1, (B) SCF, (C) c-Kit and (D) IL-4 were analyzed by semi quantitative PCR using specific forward and reverse primers. Data (means ± SD). *p<0.05, Normal Vs. 5FU and KG Vs. 5FU.

To elucidate the specific mechanisms associated with the KG-induced increase in myelopoiesis genes *in vivo*, we treated a group of mice with 5FU, followed by KG. We observed marked induction of IL-1, stem cell factor (SCF), c-Kit and IL-4 mRNA in spleen tissue (Fig. 5 A, B, C, D). Interestingly, IL-1 mRNA expression was significantly enhanced in a dose-dependent manner, following treatment with KG. Similarly, SCF and c-Kit levels were increased after KG treatment. However, IL-4 mRNA expression was not significantly increased in the presence of KG.

### Effects of KG on 5FU-induced changes in GM-CSF, IL-3 and CFU-GM

**Figure 6:**
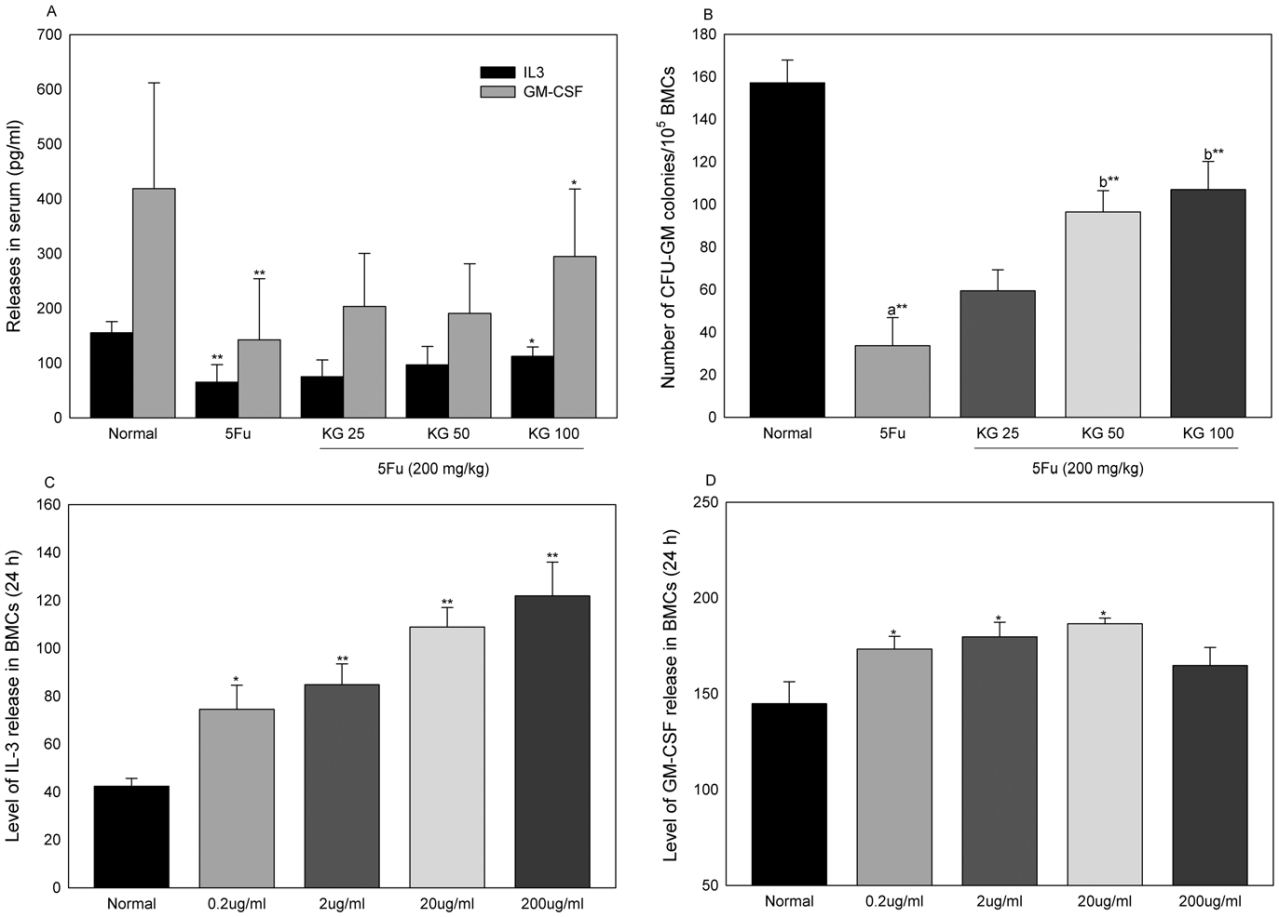
Effects of KG on 5FU induced changes IL-3, GM-CSF and CFU-GM. Serum separated from blood collected from abdominal aorta of mice was subjected to cytokine analysis using Elisa kit (A). Bone marrow cells isolated from mice were grown in MethoCult methylcellulose-based complete medium in a 5% CO2 incubator for 14 d (B). Isolated bone marrow cells cultured in 6 well plated treated with KG (0.2, 2, 20 200 µg/ml) for 24 h and conditioned media was subjected to cytokine (pg/ml) analysis using commercial kit method (C &D). Data were expressed as means ± SD. ^*^ *P*<0.05 ^**^ *P*<0.01 compared with normal, a – normal Vs. 5FU, b – KG Vs. 5FU.

Serum levels of IL-3 and GM-CSF were significantly decreased on day 7 after 5FU injection (Fig. 6A). The group of mice treated with 100 mg/kg KG showed significant increase in IL-3 and GM-CSF levels, while changes in the levels of IL-3 and GM-CSF in the presence of 25 and 50 mg/kg KG did not display statistical significance, compared to that in mice treated with 5FU alone. Granulocyte macrophage colony forming unit of bone marrow cells (CFU-GM) in the group of mice treated with 5FU was significantly lower (approximately 5-fold), compared with the normal control group, indicating reduced cellularity (Fig. 6B). Mice treated with 50 and 100 mg/kg KG displayed bone marrow reconstitution, as observed from the significant increase in GM colony forming units (by 3-fold), compared to the group treated with 5FU alone.

### Effects of KG on IL-3 and GM-CSF release in cultured bone marrow cells

To evaluate whether KG directly affects the bone marrow environment to accelerate the release of IL-3 and GM colony stimulation factor (GM-CSF), cultured bone marrow cells were treated with different doses of KG (Fig. 6 C and D). Compared with control cells, KG (0.2, 2, 20 and 200 µg/ml) promoted IL-3 release in bone marrow conditioned medium after 24 h of stimulation. The GM-CSF level was increased significantly after KG (0.2, 2 and 20 µg/ml) treatment. However, the highest dose of KG (200 µg/ml) did not induce significant release of GM-CSF, compared with control (untreated) cells.

### Effect of KG on expression of myelopoiesis genes in vitro

**Figure 7:**
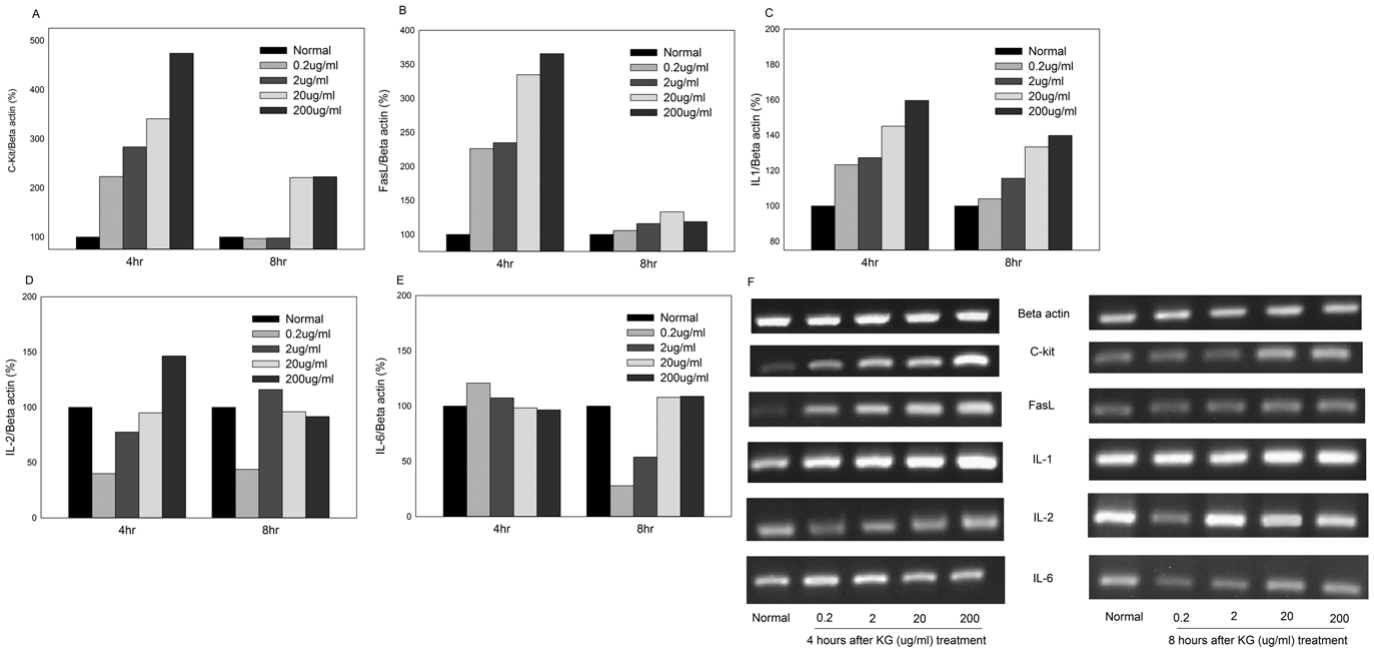
Effects of KG on genes c-Kit, FasL, IL-1, IL-2 and IL-6 in bone marrow cells. Bone marrow cells treated with KG (0.2, 2 20 and 200 µg/ml) for 4 and 8 h (n = 3). Total RNA was isolated from bone marrow cells using RNAase mini kit and analyzed by semi quantitative PCR using mouse specific primers. The relative intensities of the bands (F) were determined with the use of the ratios to β actin (normalized) and expressed as percentage (A–E).

We assessed an array of mRNA expression patterns to determine the effects of KG on cultured bone marrow cells at two time-points (4 and 8 h). KG treatment for 4 h dramatically enhanced c-Kit mRNA expression in a dose-dependent manner, while expression in mice treated with KG for 8 h was similar to that observed in untreated (normal) and 0.2 or 2 µg/ml KG-treated groups. Treatment with 20 and 200 µg/ml KG induced a 2-fold increase in the c-Kit mRNA level (Fig. 7a). FasL was significantly activated after 4 h of treatment with KG (0.2, 2 µg/ml) (>2-fold) and >3-fold with 20 or 200 µg/ml KG. After 8 h of KG treatment, dose-dependent activation was observed, but to a relatively smaller degree than untreated control cells (Fig. 7B). IL-1 was activated by 20–60% after 4 h and 8 h treatment with KG (0.2–200 µg/ml) (Fig. 7C).

**Figure 8:**
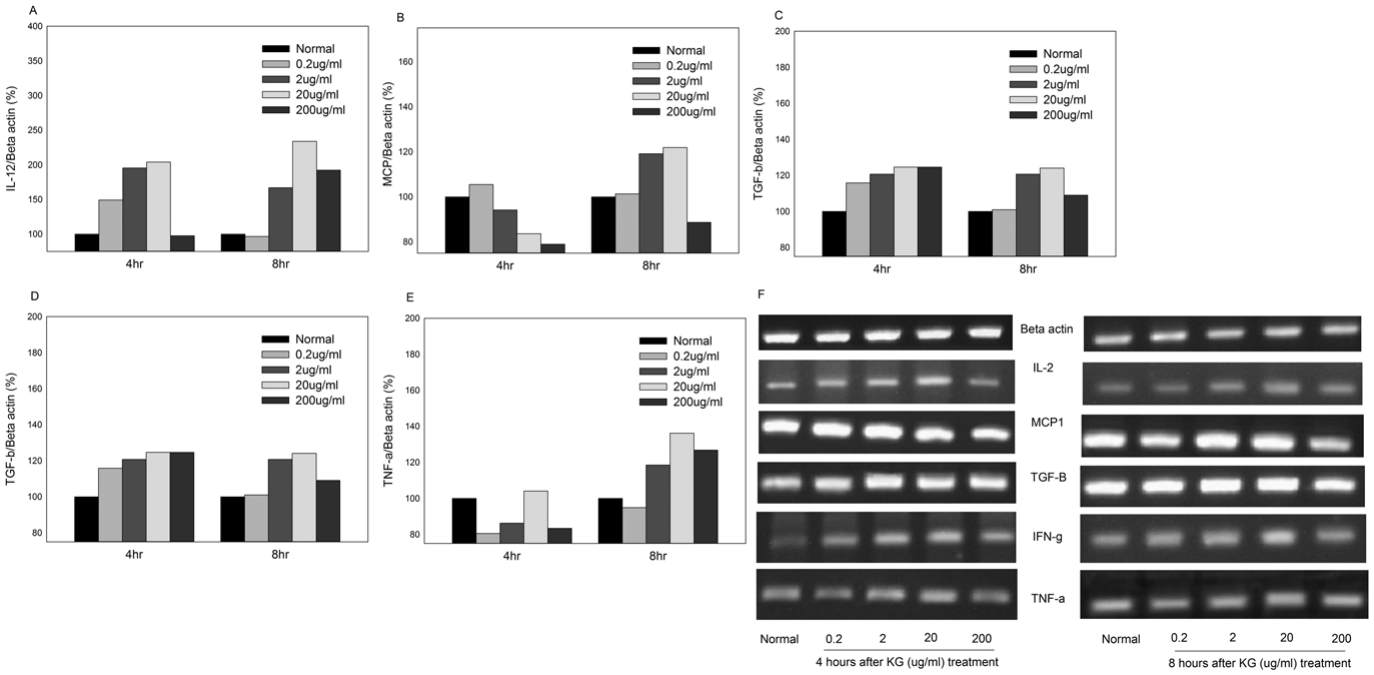
Effects of KG on genes IL-12, MCP1, TGF-β, IFN-γ and TNF-α in bone marrow cells. Bone marrow cells treated with KG (0.2, 2 20 and 200 µg/ml) for 4 and 8 h (n = 3). Total RNA was isolated from bone marrow cells using RNAase mini kit and analyzed by semi quantitative PCR using mouse specific primers. The intensities of the bands (F) were determined with the use of the ratios to β actin (normalized) and expressed as percentage (A–E).

We observed no significant upregulation of IL-2 mRNA expression after 4 and 8 h treatment with KG. Administration of 200 µg/ml KG enhanced gene expression by 0.5-fold, while treatment with lower concentrations (0.2 μg/ml) led to extremely low expression, compared to that in untreated cells (normal) (Fig. 7D). Similarly, IL-6 gene expression was slightly increased after 4 h of KG (0.2 and 2 μg/ml) treatment, but remained very low at 8 h KG treatment at these doses. KG administered at concentrations of 20 and 200 μg/ml promoted a slight increase in IL-2 after 8 h of treatment (Fig. 7E). However, we observed significant activation of IL-12 mRNA (∼0.5- to 2-fold, respectively) after 4 h of KG treatment at a range of doses (0.2–200 μg/ml). KG (0.2 μg/ml) did not significantly promote IL-12 at 8 h, but enhanced IL-12 expression at doses of 2 and 20 μg/ml (Fig. 8A). A small increase in MCP1 was observed after 4 h of treatment with KG (0.2 μg/ml), while a gradual decline was observed at the 2–200 μg/ml concentration range. After 8 h of treatment with KG (2 and 20 μg/ml), expression was enhanced by 0.2-fold, compared to that in control cells (Fig. 8B).

Expression patterns of progenitors, TGF-beta, IFN-gamma and TNF-alpha, which play a vital role in hematopoietic lineage, were further examined. After 4 h of treatment, KG (0.2–200 μg/ml) induced a >0.2-fold increase in TGF-beta expression and at 8 h (2 and 20 μg/ml KG), up to a 0.2-fold increase, compared to untreated control group (Fig. 8C). After 4 and 8 h of stimulation, IFN-g gene expression was increased by 60% and 90% in 0.2 and 20 μg/ml KG-treated groups, respectively, and by 40% in the KG (200 μg/ml) group. At the 8 h time-point, 0.2 and 2 μg/ml KG induced a 20% increase in IFN-g gene expression, 20 μg/ml KG led to a 40% decrease and 200 μg/ml KG induced a 50% decline (Fig. 8D). Similarly, the TNF-alpha level was increased by 10% after 4 h stimulation with KG 20 μg/ml KG, while the other doses did not elevate expression. Instead, a 30–40% decline in expression was observed, compared to untreated cells. At 8 h, KG (2, 20 and 200 μg/ml) led to a 20–40% increase in TNF-α, compared to untreated control cells (Fig. 8E).

### Effect of Rg1 on myelopoiesis gene expression in vitro

**Figure 9:**
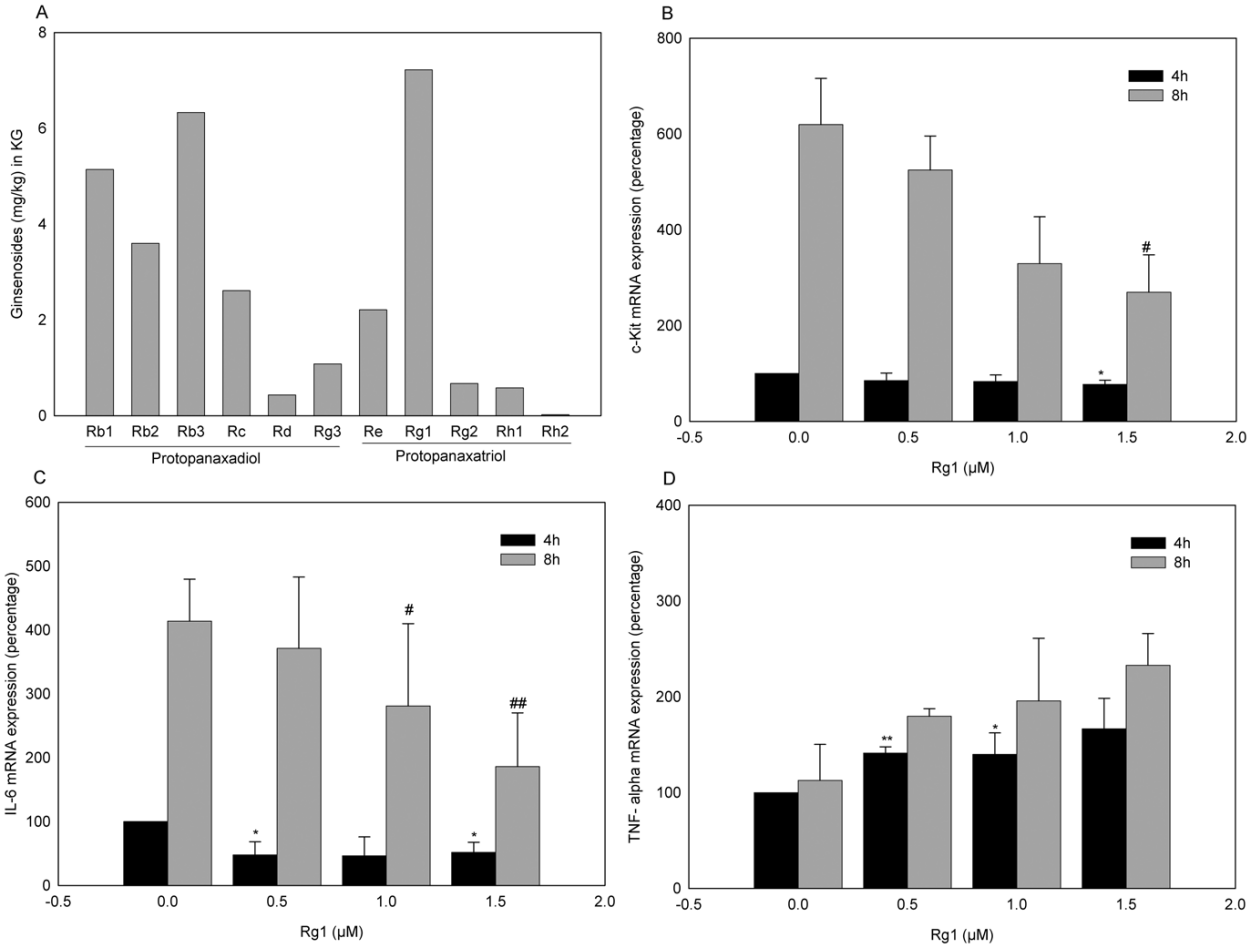
Compositional analysis of KG and role of Ginsinoside Rg1 on IL-6, c-Kit and TNF-α in bone marrow cells. Column was eluted with solvents A (18% Acetonitrite) and B (80% Acetonitrite) at flow rate of 2.5 ml/min. Solution 100% A and 0% B changing over 32 min, 80% A and 20% B to 80 min, 0% A and 100% B to 100 min, and 100% A and 0% B to 110 min were used, amount of ginsinosides present (n = 3) were expressed in mg/kg (A). (b–d) Bone marrow cultured cells were treated with Ginsinoside Rg1 (0.5, 1 and 1.5 µmol/L) for 4 and 8 h. mRNA isolated from bone marrow cells using Trireagent and products were analyzed by quantitative PCR using specific primers. Results were normalized by using the reference gene, actin, and are represented as percentage versus the reference gene. Data were expressed as means ± SD. ^*^ *p*<0.05, ^**^ *p*<0.01 compared with 4 h blank control (Untreated),^ #^ *p*<0.05, ^##^ *p*<0.01 compared with 8 h blank.

The amounts of protopanaxadiol and protopanaxatriol ginsenosides in KG extracts assessed using the high performance liquid chromatographic system (HPLC) are shown in (Fig. 9A). On the HPLC profile, Rg1 displayed the highest concentration (mg/kg). In view of this finding, we hypothesized that Rg1 plays a vital role in promoting the immunogenic role of KG. Accordingly, the mRNA expression patterns of c-kit, IL-6 and TNF-α in cultured bone marrow cells were examined at the two time-points of 4 and 8 h. Treatment with Rg1 did not affect c-Kit mRNA expression at the 4 h time-point while 1.5 µMol Rg1 induced a significant decline (Fig. 9B). IL-6 was significantly decreased after 4 h of KG (0.5, 1.5 µg/ml) and 8 h of Rg1 (1, 1.5 μMol) treatment (Fig. 9C). TNF-α was activated by 40–60% after 4 h and 70–120% after 8 h of exposure to Rg1 (Fig. 9D).

## Discussion

Hematological cytopenia resulting from chemotherapy is one of the major dose-limiting factors of cancer treatment. Attempts to reduce cytopenia, for instance, with the use of hematopoietic factors may lead to escalation of the cytotoxic dose for effective destruction of cancer cells. A commonly accepted theory is that several hematopoietic factors have the potency to shorten cytopenic intervals when administered to chemotherapy patients. Consequently, the doses of chemotherapy drugs, such as 5FU, have been increased in a number of trials to accelerate effects. However, while cell proliferation is slowly decreased, the anti-cancer effect is not significantly enhanced at high doses of 5FU [13]. Therefore, to alleviate bone marrow suppression by 5FU, natural products like ginseng are preferred for promoting hematopoiesis, proliferation and differentiation of myeloid progenitor cells. Our hematologic results showed that intraperitoneal injection of 5FU dramatically affects blood parameters, signifying severe cytopenia in mice. As neutrophil, leukocytes have the shortest half-life in circulation, their continuous renewal is essential to prevent the sequel of chemotherapeutic agent-induced neutropenia, leucopenia, and thrombocytopenia. These effects were not completely alleviated, but critical loss of total white blood cells, lymphocytes, neutrophil and platelets was significantly attenuated after treatment with KG at doses of 50 and 100 mg/kg. This accelerated hematopoietic restoration after injury indicates that KG plays a role in enhancing the proliferation of myeloid progenitors. Earlier reports have demonstrated that cyclophosphamide-induced leucopenia is effectively alleviated by the ginsenoside, Rg1 [9]. Consistent with peripheral blood analysis data, injection of 5FU induced a drastic reduction in the cellular component in bone marrow. 5FU-induced large vacuole formation and increased bone marrow cellularity were improved following KG treatment. Spleen cell damage was considerably improved after KG treatment, while recovery of thymus damage was not significant. These cumulative effects support an anti-cytotoxic effect of KG, as well as a positive role in hematopoietic lineage development.

Interleukin 1, a major cytokine, induces proliferation and differentiation of lymphocytes and neutrophil activation. IL-1 is one of the important primary factors in the biological defense mechanism that enhances the restoration of stem and progenitor subpopulations in murine marrow, accelerating hematopoietic recovery in chemotherapy induced myelosuppressed mice [14]. Similarly, SCF and CD-117 (c-Kit) are well-known major hematopoietic stimulators in chemotherapy or radiation-induced myelosuppression. A recent study showed that SCF, the ligand for the c-kit tyrosine kinase receptor, plays an important role in megakaryopoiesis and interacts with many other cytokines to promote myeloerythroid and lymphoid stem cells [15], [16]. In view of the significant elevation of serum IL-3 and GM-CSF after KG treatment, it is reasonably hypothesized that activation of SCF and its receptor stimulates proliferation and maturation of granulocytes and macrophage myeloid cells. This cytokine additionally stimulates various functions of leukocytes, including cytotoxicity of granulocytes and macrophages, phagocytic activity, and degranulation of neutrophils. IL-3 accelerates the recovery of circulating hematopoietic lineage suppressed by specific chemotherapy agents and acts as an early differentiator of stem cells [17]. This cytokine additionally stimulates the growth and effector functions of mature cells, such as macrophages and lymphocytes [18]. IL-3 and GM-CSF levels are drastically low in sera of mice injected with 5FU, indicative of a defect in the hematopoietic lineage during hematopoiesis. The observed improvements after treatment with KG (100 mg/kg) support its role in modulating early hematopoietic factors against 5FU-induced myelosuppression.

The CFU-GM content was suppressed 2-fold in 5FU-damaged bone marrow in mice compared to their untreated counterparts. KG enhanced the CFU-GM level by 3.5-fold, compared to that in mice treated with 5FU alone, indicating amelioration of side-effects, such as stimulates the differentiation and proliferation of granulocytes and monocytes in myeloid lineage progenitor cells. Earlier reports have demonstrated that *Panax notoginosides* or panaxadiol saponin increases the number of peripheral white blood cells, improves the hematopoiesis function of bone marrow, and promotes the proliferation of hematopoietic CFU-GM and CFU-E progenitors in mice with immune-mediated aplastic anemia upon bone marrow suppression [19]. The interrelationship between human health and herbal medicines is well documented. The bioactive constituents of *Panax ginseng* are believed to be saponins, and their unique chemical compositions are related to various pharmacological effects, including anti-cancer, anti-inflammatory, radioprotection, anti-emesis, immune stimulating, and anti-aging acitivites [20]. HPLC analysis has revealed the presence of high amounts of Rg1, Rb3, and Rb1 in KG. Moreover, recent studies have shown that the ginsenoside, Rg1, a steroidal saponin abundant in ginseng, is one of the most active components responsible for many of its pharmacological effects. Consistently, another study has reported that the ginsenoside, Rg3, exerts a hematopoietic effect by enhancing the proliferation of bone marrow cells and splenocytes [20], [21]. In the present investigation, we have employed a saponin-rich fraction of KG. However, the specific agent responsible for augmentation of hematopoietic stimulators is yet to be clarified.

To determine the direct effect of KG on myelopoiesis we monitored the levels of IL-3 and GM-CSF in cultured bone marrow cells treated with KG. The utility of IL-3 with the GM-CSF protocol has been confirmed by a number of *in vitro* and *in vivo* studies. Both IL-3 and GM-CSF have pleiotropic effects on hematopoietic cells and exhibit overlapping activities [22]. However, IL-3 targets the earliest progenitors to induce their proliferation, differentiation, survival, and progeny, while GM-CSF is a hematopoietic cytokine with the ability to stimulate proliferation and maturation of granulocyte and macrophage myeloid cells [23]. The results from these analyses suggest a role of KG in enhancing the production of hematopoietic factors and proliferation of hematopoietic progenitor cells for the recovery of mice from 5FU-induced leucopenia or improved cytokine release indicates that KG enhances cytokine production by lymphocytes. One report suggested that total saponin of *panax ginseng* promotes hematopoietic factors and induces endothelial cells and monocytes in the microenvironment to synthesize and secrete GM-CSF, either through a direct or indirect route [24]. Consistently, reports have shown that *Panax notoginosides* acts as a growth factor or displays synergistic efficacy with other growth factors (such as stem cell factor, GM-CSF and IL-3) in the proliferation of hematopoietic stem/progenitor cells [25], [26].

In recent decades, the discovery of new natural noble agents has been extensively pursued, with a view to reducing the deleterious side effects of chemotherapy. Recombinant cytokines are well-characterized as immune modulators [27]–[29] and have been applied in immunotherapy for a variety of cancers. However, these compounds tend to be too toxic with very short half-lives, suggesting that their endogenous induction is a more effective immunotherapeutic approach in cancer treatment and improving chemotherapy-related side-effects [30]. To further determine the mechanism underlying the anti-myelosuppression activity of KG, we analyzed the relevant myelopoiesis-promoting factors in cultured bone marrow cells. Factors, such as c-kit, FasL, IL-1, IL-2, and IL-6, are involved in various stages of development of granulocyte-monocyte/macrophage lineage and hematopoietic stimulators [31]. The ability of SCF to protect megakaryocytic progenitors from chemotherapy-induced myelosuppression signifies a link between c-kit signaling and thrombocytopenia that may influence the extent of megakaryocyte loss during chemotherapy. Several pharmacologic agents that inhibit c-kit frequently induce megakaryocyte loss and thrombocytopenia. Thrombocytopenia is particularly evident when 5FU is combined with other chemotherapeutic drugs, suggesting that interactions between c-kit and endogenously produced SCF are important for megakaryocyte survival in myelosuppressive conditions. IL-1 affects hematopoiesis via different mechanisms [32]. The enhanced c-kit and IL-1 expression in bone marrow cells suggests that KG is more effective if administered simultaneously with chemotherapeutic treatment and during the entire duration of the chemotherapy cycle to prevent the occurrence of drug-induced myelosuppression. However, cultured bone marrow cells treated with Rg1 alone did not enhance c-kit expression. Ginsenosides present in the KG may have diverse as well as synergistic actions, and therefore the possibility of other active principles in KG extracts contributing to immunogenic activity cannot be ruled out at this stage.

Interleukin 6 (IL-6) is a cytokine produced by a number of normal and transformed cell lines [33]. IL-6 can either promote or inhibit the growth of tumor cells, depending on the cell type. IL-6 acts in concert with IL-3 to induce multilineage progenitors from murine spleens and M-CSF to stimulate the number and size of CFU-M [34]. In our experiments, no significant activation of IL-6 was observed following 4 h and 8 h treatment with KG or Rg1, in contrast to earlier reports demonstrating that ginsan elevates IL-6 mRNA expression in animal models [35], [36]. Hematopoiesis is a diverse process regulated by various hematopoietic growth factors. In addition to IL-6, other factors, such as IL-1, GM-CSF, IL-3 and stem cell factor, may act on early hematopoietic lineages. However, there is no conclusive evidence showing a direct association of KG with protection against myelotoxicity or hematopoiesis [37].

IL-2 was primarily explained as a mitogenic signal and growth factor for T lymphocytes [38]. The findings of differential expression of IL-2R during *in vitro* differentiation of human myeloid cells and increase in the number of colony-forming unit-granulocyte/macrophage (CFU-GM) progenitor cells after treatment of cancer patients with IL-2 are consistent with its positive effect on myelopoiesis. However, other studies have reported contradictory findings [39]. In the absence of IL-2, mice develop a profound hematopoietic disorder characterized by defective myelopoiesis and decreased number of mature granulocytes. We obtained conflicting data on IL-2 expression in our experiments [40]. Low concentrations of KG did not enhance IL-2 gene expression, while the highest dose induced a considerable increase in expression. A dose of 20 µg/ml KG-induced slight activation following 8 h of treatment, but not the other concentrations. Considering the link between CFU-GM and IL-2, we examined the effect of KG on CFU-GM expression. No inhibition of the CFU-GM level was evident. Although serum IL-2 was not monitored, we propose that depending on the cell type, KG promotes myelopoiesis without affecting colony forming units. However, further experiments are necessary to clarify its mechanism of action.

Fas and FasL influence human hemopoietic progenitor cell numbers in different *in vitro* systems [41]. Until recently, studies on the involvement of Fas and FasL in hemopoiesis have focused on their proapoptotic functions. However a number of reports indicate that activated downstream caspases can exert regulatory effects in the absence of cell death. Fas, FasL, and caspase activation are likely to play an important role in the regulation of myelopoiesis [42]. FasL was significantly elevated after 4 and 8 h treatment with KG, this observation leads to a hypothetical hypothesis that regulation of myelopoiesis may be via non-apoptotic pathway. Earlier reports have shown that *panax notoginseng* not only stimulates cell proliferation but also inhibits the activity of caspases and apoptosis of hematopoietic cells [9], [26].

Specific cytokines, including IL-1 and IL-12, stimulate the precursors of bone marrow cells and display radioprotective and chemoprotective activities [43], [44]. Our results clearly showed that KG increases IL-12 expression. It is believed that the chemoprotective effects of KG on bone marrow cells are mediated through IL-12. Consistently, a recent report showed enhancement of IL-12 expression by the acidic polysaccharide of ginseng. Enhancement of IL-12 within the tumor environment has been shown to contribute to tumor clearance through a variety of mechanisms, including restoration of the cytotoxicity of tumor-resident CD8+ T-cells [8]. Therefore, IL-12 activation after KG treatment may play a positive role in cancer treatment strategies, particularly chemotherapy. MCP1 represents a family of cytokines that were originally designated on the basis of their activity as chemo-attractant cytokines for mature blood cells. MCP1s have been implicated in the control of myelopoiesis, especially as negative regulators [45]. In our experiments, 4 h treatment with KG decreased MCP1 expression, while a small increase was evident upon 8 h treatment. Ginseng exerts a significant inhibitory activity on MCP1, leading to reduced leukocyte infiltration and inflammatory response. Elevated levels of MCP1 following 8 h of KG treatment may correlate with the earlier report that endogenous chemokines cooperating with other growth factors inhibit cycling of primitive normal progenitors in long-term culture. However, its precise role is yet to be established.

There are no documented studies on the effects of KG on specific hematopoietic inhibitors, such as TGF-β and IFN-γ, in the literature. Activation of certain inhibitors correlates with hypothesize that some cytokines may accelerate the haematopoietic development, and the inhibitors like IFN-γ, TNF-alpha may keep the haematopoietic stem cell in a quiescent state to protect them from exhaustion or from the cytotoxic effect of chemotherapy drugs [46]. Generally, endothelial cells elaborate TGF-β, which consequently has an inhibitory effect on progenitor growth. However, research has shown that TGF not only activates CD34 antigen before S phase entry but also maintains a high level of CD34 expression on cells that have escaped cell cycle inhibition. These lines of evidence suggest that TGF acts as a vital physiological factor ensuring the maintenance of a stem cell reserve [47], [48]. However, neither of these cytokines was found to be solely responsible for the inhibitory effect on GM-CFC growth; rather, the effect was partly caused by synergism between the two factors, and most likely by other factor(s) that are yet to be identified [46]. While our experiments showed that TGF-β is significantly activated upon KG treatment, the precise mechanism underlying this activation is yet to be established.

Hematosuppression, one of the most severe side-effects of chemotherapy, may lead to immunological inadequacy, severe infection, discontinuation of treatment, or even death. There is evidence to support beneficial roles of IFN-γ and GM-CSF in protective immunity against infection [49]. From this viewpoint, IFN-γ may play a positive role in cancer patients who often encounter opportunistic infections. However, there have been conflicting reports on the positive of IFN-γ regulatory effect on myelopoiesis. Ginsenosides, such as Rg3 and Rh2, have been shown to promote anti-cancer effects and reduce side-effects during chemotherapy with cyclophosamide [50]–[52].

TNF is a potent inhibitor of erythropoiesis that suppresses the growth of erythroid and myeloid progenitors *in vitro*. These cytokines, in turn, affect the differentiation of early bone marrow progenitor cells by altering their response to CSFs [53]. However, earlier report has shown that TNF-alpha is an important mediator of the granulocyte-macrophage mature cell lineage, which increases their phagocytic, cytotoxic, and bacteriocidic properties [54]. Moreover, TNF-alpha accelerates the release of granulocytes from bone marrow. The role of TNF-alpha in regulation of myelopoiesis has been not extensively elucidated [55].

An earlier report demonstrated that ginsan stimulates the expression of TNF-α and major histocompatibility complex (MHC) class II molecules in murine dendritic cells and proliferation of allogeneic CD4 (+) T lymphocytes [8]. Our results showed that KG modulates TNF-α expression. Specifically, 4 h of KG treatment has a suppressive effect, while 8 h treatment leads to significant activation. Recent reports have additionally shown that Fas expression on hematopoietic progenitor cells is induced by cytokines, such as IFN-γ and TNF-α [41]. These results favor the hypothesis that KG or Rg1 also stimulates macrophages to produce cytokines (IL-1, TNF-alpha), which may, in turn, induce the release of growth factor through lymphocytes, several tissues and organs, including cells of the bone marrow microenvironment [56].

The data collectively indicate that KG improves bone marrow cellular toxicity in 5FU-induced mice and *in vitro* myelopoiesis, and accelerates hematopoietic recovery by modulating the release of cytokines. The ginsinoside, Rg1, a major constituent of KG, directs the immunogenic property of Korean ginseng to some extent.

## Materials and Methods

### Preparation of KG and HPLC analysis

Korean *P. ginseng* root was obtained from Ginseng Nonghyup (Keum-san, Korea). Sliced ginseng roots (1.2 kg) were boiled in 6.5 l of water for 100 min at 120^°^C using a high-speed automatic non-pressure earthen pot (Kyung-Hee Co-operation, Seoul, Korea), and the extraction procedure repeated with 4.5 l of water. Then, KG was centrifuged for 30 min at 1,500 rpm, and the supernatant lyophilized using a vacuum-freeze drying system and stored at –40°C. Limulus amebocyte lysate (LAL) assay was performed to confirm endotoxin levels. The amount of protopanaxadiol and protopanaxatriol ginsenosides in KG was estimated using High performance liquid chromatographic system (HPLC). The HPLC system consisted of a Waters Alliance 2695 HPLC pump, Waters Alliance 2695 Auto sampler, and Waters 996 PDA (United States, Connecticut). HPLC conditions were according to a previously described method (Raghavendran et al., 2010). GinsenosideRg1was purchased from Wako Pure Chemical (Osaka, Japan) with 98% HPLC purity.

**Table 1:** Primers used for semi-quantitative PCR.

Genes	Forward primer	Reverse primer	Product size (bp)
C-Kit,	GAACAGGACCTCGGCTAACA	GCCATTTATGAGCCTGTCGT	220
FasL,	ACTCCGTGAGTTCACCAACC	CAAGACTGACCCCGGAAGTA	267
IL-1,	AAG CTC TCC ACC TCA ATG GA	TGC TTG AGA GGT GCT GAT GT	302
IL-2,	TGC TCC TTG TCA ACA GCG	TCA TCA TCG AAT TGG CACTC	391
IL-6,	TGTGCAATGGCAATTCTGAT	TGGTCCTTAGCCACTCCTTC	357
IL-12,	ATGTGGGAGCTGGAGAAAGA	TGGCCAGCATCTAGAAACTCT	194
MCP1,	ATGCAGGTCCCTGTCATG	GCTTGAGGTGGTTGTGGA	479
TGF-beta,	TGAGTGGCTGTCTTTTGACG	TTCTCTGTGGAGCTGAAGCA	310
IFN-g	GGA TAT CTG GAG GAA CTG GC	GAG CTC ATT GAA TGC TTG GC	250
TNF-a	CTCCCAGGTTCTCTTCAAGG	TGGAAGACTCCTCCCAGGTA	195
SCF	CCTCTTGTCAAAACCAAGGAG	GGCCTCTTCGGAGATTCTTT	329
IL-4	TCAACCCCCAGCTAGTTGTC	GCATGGAGTTTTCCCATGTT	254
βactin	GTG GGG CGC CCC AGG CAC CA	CTC CTT AAT GTC ACG CAC GAT TTC	539

### Animals

Male C57BL/6, (Orientbio, Gyeonggido, Korea) weighing between 18±20 g were used in this study. Mice were housed in standard polypropylene transparent cages under environmentally controlled conditions (Temperature, 22±2°C; Humidity, 55–60 %) with a 12 h light: 12 h dark cycle (light on 09:00 and 21:00 h). Mice were fed commercial standard chow (Samtako, Osan, Korea) and tap water *ad libitum*. 5FU-induced myelotoxicity experiments were designed and performed in strict accordance with the regulations for laboratory animal care (NIH publication No. 85-23, revised 1985), approved by the Institutional Animal Care and Use Committee of Daejeon University (DJUARB 2010–017).

### Experimental design

After one week acclimatization all mice were randomly divided into normal (n = 13), 5FU (200 mg/kg) alone (n = 13), and KG (25, 50 and 100 mg/kg, orally) treatment (n = 13/each dose). Except normal all the other groups were given 5FU as intraperitoneal injection on 0^th^ day and after 24 h, 39 mice were post treated with KG for 6 consecutive days. For hematology 8 mice from each group were used to check the optimal effect of KG and 5 animals were sacrificed on day 7 for histopathology, cytokines and gene expression studies, for colony forming assay (n = 3 mice/group) were used (Fig. 1).

### Hematology

Peripheral blood was collected from the retro-orbital venous plexus with an EDTA-coated capillary tube at different time points (0, 3, 5, 7, 10 and 14 Days). The red blood cells (RBC), hemoglobin (Hb), white blood cells (WBC), lymphocytes and neutrophils, hematocrit (HCT), platelets in each sample were measured by a HEMA VET 850 automatic analyser (CDC Technologies, CT, USA).

On the experimental day (7^th^) animals were sacrificed under ether anesthesia and blood was collected from the abdominal aorta. A portion of the blood was used for red blood cells (RBC), hemoglobin (Hb), hematocrit (HCT), platelets, white blood cells (WBC), lymphocytes and neutrophils estimation using an HEMA VET 850 automatic analyser (CDC Technologies, CT, USA). Another portion of the blood was subjected to serum separation and stored at –70°C for cytokine analysis.

### Measurement of cytokine levels and histopathological analysis

Enzyme-linked immuno-sorbent assay (ELISA) kits were purchased from BD Biosciences (San Diego CA, USA) for assay of cytokines. The concentrations of IL-3 and GM-CSF in serum (7^th^ day) were examined. A standard curve was run on each assay plate using recombinants of the respective cytokines in serial dilutions.

Spleen, thymus and right femur from all mice were fixed in 10% buffered formalin, and the femur was decalcified with Calci-Clear Rapid (National Diagnostics, Atlanta, GA, USA). Spleen, thymus and femur were then embedded in paraffin, sectioned at 4 µm, and stained with hematoxylin-eosin. Representative microphotographs were obtained with a light microscope (Leica, Wetzlar, Germany).

**Table 2:** Primers used for quantitative RT-PCR.

Genes	Forward (5′-3′)	Reverse (5′-3′)
Actin	ACCGTGAAAAGATGACCCAG	TCTCAGCTGTGGTGGTGAAG
c-kit	TGTGTCTACATCCGTGAACTCCAT	AGCGTCTCCTGGCGTTCATA
IL-6	GACTTCCATCCAGTTGCCTTCTT	TCCACGATTTCCCAGAGAACA
TNF-α	CGTCGTAGCAAACCACCAAGT	TTGAAGAGAACCTGGGAGTAGACA

### Extraction of total RNAs from spleen and PCR

The spleen tissues (100 mg) were homogenized with RIPA buffer and centrifuged at 10,000×*g* for 15 min at 4°C. The supernatant fraction was used for total RNAs extraction using RNAase midi kit (Qiagen, Valencia, CA, USA). Sixteen microliters of double distilled water mixed with 1 µl of forward and reverse primers and mixture was added to PCR pre mix (Bioneer, Korea). Then 3 µl of cDNA was added to each tube. PCR amplification was carried out using a protocol initial denaturation temperature of 94 degree for 10 minutes followed by 30 cycle's amplification at (94 degree 1 min, 60 degree 40 s, 72 degree 40 s). with an additional extension at 72°C for 10 min. The PCR products were fractionated on a 1% agarose gel and visualized by ethidium bromide staining. The band intensity of ethidium bromide fluorescence was measured using NIH Image Analysis Software Version 1.61 (National Institutes of Health, Bethesda, MD). The nucleotide sequences of forward and reverse primers used for PCR are shown in Table 1.

### Isolation of bone marrow cells

Bone marrow stem cells were harvested from the femurs of C57BL/6 male mice. Bones were briefly immersed in 70% ethanol (3 s), and rinsed four times (2 minutes each) in Phosphate Buffer Solution containing antibiotics (penicillin-streptomycin) under sterile conditions. The epiphyses of each bone were removed with a razor blade, and the marrow was flushed from the diaphysis with a syringe and 26.5-gauge needle and collected in DMEM (Welgene, Daegu, Korea) containing 10% (v/v) fetal bovine serum (FBS, Welgene) and 1% penicillin-streptomycin mixture (Welgene). The marrow cell suspension was gently drawn through an 18-gauge needle to mechanically dissociate the mixture into a uniform single cell suspension. Nucleated cells were counted using a blood cell counter (HEMAVET; CDC Technologies)

### Colony-forming and cytokine assay

To examine the effects of KG on bone marrow stem cells, C57BL/6 mice were injected intraperitoneally with 5FU (0.2 g/kg). Mice were given KG (25, 50 and 100 mg/kg) or distilled water (for naive and control groups) for 6 consecutive days after the 0^th^ day of 5FU injection. After thoroughly mixing the nucleated cells (1×10^5^) with 4 mL MethoCult methylcellulose-based complete medium (Stem Cell Technologies, Seattle, WA, USA), media (1.1 mL per dish in triplicate) were cultured in a 5% CO2 incubator for 14 d. According to the morphological characteristics, the number of colonies assessed by CFU-GM was counted under an inverted microscope.

Bone marrow cells isolated from C57BL/6 male mice were seeded in a six-well culture plate (1×10^5^ cells per well), and treated with or without KG, Normal (untreated control cells) 0.2, 2, 20 and 200 µg/ml in a 5% CO2 incubator. After 24 h of incubation with KG, conditioned medium of bone marrow cells were collected and subjected to cytokine analysis (IL-3 and GM-CSF) using BD Biosciences (San Diego CA, USA) for assay of cytokines. A standard curve was run on each assay plate using recombinants of the respective cytokines in serial dilutions.

### Semi quantitative and Quantitative PCR

Bone marrow cells were seeded in a six-well culture plate (5×10^6^ cells per well), and treated with or without KG Normal (untreated control cells), 0.2, 2, 20 and 200 µg/ml) for two points 4 and 8 h. After purification of total RNA using an RNAase mini kit (Qiagen, Valencia, CA, USA). To determine the expression pattern of all mRNA, 1 µL of cDNA was amplified by a thermal cycler (TaKaRa, Japan) using the specific primers. The PCR mixture was made as follows; 1.5 U of *Taq* DNA polymerase (Bioneer, Korea), 3 µL of dNTPs 10 mmol/L, 3 µL of 10×PCR buffer, 1 µL of 10 pmol forward and reverse primers, and 1 µL of cDNA in 19.7 µL of ultra distilled water. PCR amplification was carried out using a protocol of initial denaturing step at 95°C for 10 min, then 32 cycles of amplification (at 95°C for 1 min, at 60°C for 40 s, and at 72°C for 40 s ), and additional extension at 72°C for 10 min. The PCR products were fractionated on a 1% agarose gel and visualized by ethidium bromide staining. The band intensity of ethidium bromide fluorescence was measured using NIH Image Analysis Software Version 1.61 (National Institutes of Health, Bethesda, MD). The intensities of the bands were determined with the use of the ratios to β actin (normalized) and expressed as percentage. The nucleotide sequences of forward and reverse primers used for PCR are shown in Table 1.

Isolated bone marrow stem cells were plated at 5×10^6^ cells/well in 6-well plate for overnight, and treated with 0.5∼1.5 µM concentrations of Rg1 for 4 h, 8 h, and Normal (untreated control cells) for 24 h respectively. The levels of mRNA of C-kit, IL-6 and TNF-α, were determined by quantitative real-time RT-PCR. Briefly, total RNA was extracted from BMSCs using TriReagent (Molecular Research Center, Cincinnati, OH). The cDNAs were then synthesized using 2 µg of RNA template in a 20 µl reaction using the High-Capacity cDNA Reverse Transcription Kit (Ambion, Austin, TX). PCR amplifications were quantified using the SYBRGreen supermix reagent (Bio-Rad, CA) according to the manufacturer's protocol. All primer (Table 2) sets produced amplicons of the expected size, and their identities were also verified by sequencing. The cycling conditions were 95°C for 10 min, followed by 40 cycles of 95°C for 10 s, 60°C for 20 s, and 72°C for 33 s. To detect and eliminate possible primer-dimer artifacts, a dissociation curve was generated by adding a cycle of 95°C for 15 s, 60°C for 1 min, and 95°C for 15 s. Results were normalized by using the reference gene, actin, and are represented as percentage versus the reference gene Table 2.

### Statistical analysis

Data analysis was performed using the Statistical Package for Social Science (SPSS for Windows, version 10.0, 1999, SPSS Inc, Chicago, USA). Data are expressed as means ± SD. We used one-way ANOVA followed by Least Significance Deviation method and the differences were considered significant if P<0.05 P<0.01

## References

[1] Lund JE (2000). Toxicologic effects on blood and bone marrow, in Schalm's Veterinary Hematology, 5th edition (B..

[2] Ryan DH (2001). Examination of the blood..

[3] Grem JL (2000). 5-Fluorouracil: forty-plus and still ticking. A review of its preclinical and clinical development.. Invest New Drugs.

[4] Tomiak A, Vincent M, Kocha W, Taylor M, Winquist E (2000). Standard dose (Mayo regimen) 5-fluorouracil and low does folinic acid: prohibitive toxicity?. Am J Clin Oncol.

[5] Jodrell DI, Stewart M, Aird R, Knowles G, Bowman, A (2001). 5-Fluorouracil steady state pharmacokinetics and outcome in patients receiving protracted venous infusion for advanced colorectal cancer.. Br J Can.

[6] Attele AS, Wu JA, Yuan CS (1999). Ginseng pharmacology: Multiple constituents and multiple actions.. Biochem Pharmacol.

[7] Yance DR, Sagar SM (2006). Targeting angiogenesis with integrative cancer therapies.. Integ Can Therap.

[8] Kim HJ, Kim MH, Byon YY, Park JW, Jee Y (2007). Radioprotective effects of an acidic polysaccharide of *Panax ginseng* on bone marrow cells.. J Vet Sci.

[9] Zhang QH, Wu CF, Duan L, Yang JY (2008). Protective effects of total saponins from stem and leaf of *Panax ginseng* against cyclophosphamide-induced genotoxicity and apoptosis in mouse bone marrow cells and peripheral lymphocyte cells.. Food Chem Toxicol.

[10] Lu XZ, Wang JH, Wu X, Zhou L, Wang L (2008). Ginsenoside Rg1 promotes bone marrow stromal cells proliferation *via* the activation of the estrogen receptor-mediated signaling pathway.. Acta Pharmacol Sin.

[11] Leung KW, Pon YL, Wong RN, Wong AS (2006). Ginsenoside-Rg1 induces vascular endothelial growth factor expression through the glucocorticoid receptor-related phosphatidylinositol 3-kinase/Akt and betacatenin/T-cell factor-dependent pathway in human endothelial cells.. J Biol Chem.

[12] Lee YJ, Chung E, Lee KY, Lee YH, Huh B (1997). Ginsenoside- Rg1, one of the major active molecules from Panax ginseng, is a functional ligand of glucocorticoid receptor.. Mol Cell Endocrin.

[13] De Haan G, Donte B, Engel C, Loeffler M, Nijhof W (1996). Prophylactic Pretreatment of Mice with Hematopoietic Growth Factors Induces Expansion of Primitive Cell Compartments and Results in Protection against 5-Fluorouracil -Induced Toxicity.. Blood.

[14] Dinarello CA, Savage N (1989). Interleukin-1 and its biologically related cytokines.. Adv Immunol.

[15] Fackler MJ, Krause DS, Smith OM, Civin CI (1995). Full-length but not truncated CD34 inhibits hematopoietic cell differentiation of M1 cells.. Blood.

[16] Almeida-Porada G, Ascensao IL (1996). Isolation, characterization and biologic features of bone marrow endothelial cells.. J Lab Clin Med.

[17] Nand S, Sosman J, Godwin JE, Fisher RI (1994). A phase I/II study of sequential interleukin-3 and granulocyte-macrophage colony-stimulating factor in myelodysplastic syndromes.. Blood.

[18] Jones AT, Ziltener HJ (1993). Enhancement of the biologic effects of interleukin-3 in vivo by anti-interleukin-3 antibodies.. Blood.

[19] Gilmore GL, DePasquale DK, Fischer BC, Shadduck RK (1995). Enhancement of monocytopoiesis by granulocyte colony-stimulating factor.. Exp Hematol.

[20] Lee DW Y, Lau ASY (2011). Effects of *Panax ginseng* on Tumor Necrosis Factor-α-Mediated Inflammation: A Mini-Review.. Molecules.

[21] Joo SS, Won TJ, Kim MS, Lee DI (2004). Hematopoietic effect of ginsenoside Rg3 in ICR mouse primary cultures and its application to a biological response modifier.. Fitoterapia.

[22] Koike K, Ogawa M, Ihle JN, Miyake T, Shimizu (1987). Recombinant murine granulocytemacrophage (GM) colony-stimulating factor supports formation of GM and multipotential blast cell colonies in culture: comparison with the effects of interleukin-3.. J Cell Physiol.

[23] Leary AG, Yang YC, Clark SC, Gasson JC (1987). Ogawa M. Recombinant gibbon interleukin 3 supports formation of human multilineage colonies and blast cell colonies in culture: comparison with recombinant human granulocyte-macrophage colony-stimulating factor.. Blood.

[24] Chen D, Wang SL, Wang YP (2003). Experimental study on expression of GM-CSF from human endothelial cells and monocytes induced by total saponins of *Panax ginseng*.. Zhongguo Zhong Xi Yi Jie He Za Zhi.

[25] Gao RL, Chen XH, Lin XJ, Qian, XD, Xu WH (2007). Effects of notoginosides on proliferation and upregulation of GR nuclear transcription factor in hematopoietic cells.. Acta Pharmacol Sin.

[26] Chen XH, Gao RL, Zhen ZY, Qian XD, Xu WH (2006). Expression of apoptosis-related proteins in the human bone marrow hematopoietic cells treated by Panax Notoginosides.. Zhongguo Shi Yan Xue Ye Xue Za Zhi.

[27] Leonard JP, Quinto CM, Kozitza MK, Neben TY, Goldman SJ (1994). Recombinant human interleukin- 11(rhIL-11) multilineage hematopoietic recovery in mice after a myelosuppressive regimen of sublethal irradiation and carboplatin.. Blood.

[28] Paquette RL, Zhou JY, Yang YC, Clark, SC, Koeffler HP (1988). Recombinant gibbon interleukin-3 acts synergistically with recombinant human G-CSF and GM-CSF in vitro.. Blood.

[29] Winton EF, Srinivasiah J, Kim BK, Hillyer CD, Strobert EA (1994). Effect of recombinant human interleukin-6 and rhIL-3 on hematopoietic regeneration as demonstrated in a nonhuman primate chemotherapy model.. Blood.

[30] Saif MW (2005). Capecitabine versus continuous-infusion 5-fluorouracil for colorectal cancer: a retrospective efficacy and safety comparison.. Clin Col Can.

[31] Davis TA, Robinson DH, Lee KP, Kessler SW (1995). Porcine brain microvascular endothelial cells support the in vitro expansion of human primitive hematopoietic bone marrow progenitor cells with a high replanting potent: requirement for cell-to-cell interactions and colony-stimulating factor.. Blood.

[32] Zeuner A, Signore M, Martinetti D, Bartucci M, Peschle C (2007). Chemotherapy-Induced Thrombocytopenia Derives from the Selective Death of Megakaryocyte Progenitors and Can Be Rescued by Stem Cell Factor..

[33] Bartold PM, Haynes DR (1991). Interleukin-6 production by human gingival fibroblasts.. J Periodon Res.

[34] Ikebuchi K, Wong GG, Clark SC, Ihle JN, Hirai Y (1987). Interleukin-6 enhancement of interleukin-3-dependent proliferation of multipotential hemopoietic progenitors.. Proc Natl Acad Sci.

[35] Chang YS, Seo EK, Gyllenhaal C, Block KI (2003). Panax ginseng: a role in cancer therapy?. Integrat Cancer Therap.

[36] Yun TK (2003). Experimental and epidemiological evidence on nonorgan specific cancer preventive effect of Korean ginseng and identification of active compounds.. Mut Res.

[37] Rafii S, Shapiro F, Pettengell R, Ferris B, Nachman RL (1995). Human bone marrow microvascular endothelial cells support long-term proliferation and differentiation of myeloid and megakaryocytic progenitors.. Blood.

[38] Smith K (1998). Interleukin 2: Inception, impact, and implications.. Science.

[39] Metcalf D (1989). The molecular control of cell division, differentiation commitment and maturation in haemopoietic cells.. Nature.

[40] Reya T, Contractor NV, Couzens MS, Wasik MA, Emerson SG (1998). Abnormal Myelocytic Cell Development in Interleukin-2 (IL-2)–Deficient Mice: Evidence for the Involvement of IL-2 in Myelopoiesis.. Carding Blood.

[41] Alenzi FQ, Al-Ghamdi SM, Tamimi WG, Al-Sebiany AM, El-Nashar IM (2005). Apoptosis Role of FAS/FAS Ligand System in the Regulation of Myelopoiesis.. Yale J Biol Med.

[42] De Maria R, Zeuner A, Eramao A, Domenichelli C, Bonci D (1999). Negative regulation of erythropoiesis by caspase-mediated cleavage of GATA-1.. Nature.

[43] Dalmau SR, Freitas CS, Savino W (1997). Radio- and chemoprotection of bone marrow cells by opposite cell cycle-acting cytokines.. Leukemia Res.

[44] Smith JW, Urba WJ (1992). The toxic and hematologic effects of interleukin-1 alpha administered in a phase I trial to patients with advanced malignancies.. J Clin Oncol.

[45] Cashman JD, Eaves CJ, Sarris AH, Eaves AC (1998). MCP-1, not MIP-1a, is the endogenous chemokine that cooperates with TGF-b to inhibit the cycling of primitive normal but not leukemic (CML) progenitors in Long-term human marrow cultures.. Blood.

[46] Deonarain R, Verma A, Porter AC, Gewert DR, Platanias LC (2003). Critical roles for IFN-gamma in lymphoid development, myelopoiesis, and tumor development: Links to tumor necrosis factor alpha.. Proc Nat Acad Sci.

[47] Li WM, Huang WQ, Huang YH, Jiang DZ, Wang QR (2000). Positive and negative haematopoietic cytokines produced by bone marrow endothelial cells.. Cytokine.

[48] Batard P, Monier MN, Fortunel N, Ducos K, Sansilvestri-Morel P (2000). TGF-b1 maintains hematopoietic immaturity by a reversible negative control of cell cycle and induces CD34 antigen up-modulation.. J Cell Sci.

[49] Vento S, Cainelli F, Temesgen Z (2008). Lung infections after cancer chemotherapy.. Lancet Oncol.

[50] Kang XM, Zhang QY, Tong DD, Zhao W (2005). Experimental study on anti-angiogenesis in mice with Lewis lung carcinoma by lowdose of cyclophosphamide combined with ginsenoside Rg3.. Zhongguo Zhong Xi Yi Jie He Za Zhi.

[51] Li HB, Liu WL, Zhang QY, Kang XM, Zhao WH (2006). The efficacy analysis of low-dose cyclophosphamide combined with gimenoside Rg3 on advanced non-small cell lung cancer.. J Pract Oncol.

[52] Chen MW, Yao Y, Shi ZH (2005). Observation of curalive effect of combined 20(R)-ginsenoside Rg3 with chemotherapy on non-small – cell lung cancers.. Mil Med J South China.

[53] Roodman GD, Bird A, Hutzler D, Montgomery W (1987). Tumor necrosis factor-alpha and hematopoietic progenitors: effects of tumor necrosis factor on growth of erythroid progenitors CFU-E and BFU-E and hematopoietic cell lines K562, HL60, and HEL cells.. Exp Hematol.

[54] Artym J, Zimecki M (2007). The Effects of Lactoferrin on Myelopoiesis: Can we resolve the Controversy?. Postepy Hig Med Dosw.

[55] Voog E, Bienvenu J, Warzocha K, Moullet I, Dumontet C (2000). Factors That Predict Chemotherapy-Induced Myelosuppression in Lymphoma Patients: Role of the Tumor Necrosis Factor Ligand-Receptor System..

[56] Shi AW, Wang XB, Lu FX, Zhu MM, Kong XQ (2009). Ginsenoside Rg1 promotes endothelial progenitor cell migration and proliferation.. Acta Pharmacol Sin.

